# Do animation videos increase participation in national health surveys? A randomised controlled trial

**DOI:** 10.1186/s12874-023-02005-4

**Published:** 2023-08-14

**Authors:** Anne Illemann Christensen, Cathrine Juel Lau, Hanne Stald Poulsen, Ola Ekholm

**Affiliations:** 1 National Institute of Public Health, University of Southern Denmark, Copenhagen, Denmark; 2 Center for Clinical Research and Prevention, Bispebjerg and Frederiksberg Hospital, Capital Region of Denmark, Copenhagen, Denmark; 3 Data & Development Support, Region Zealand, Sorø, Denmark

**Keywords:** Data collection, Health surveys, Non-response, Animation, Questionnaires, Survey methods

## Abstract

**Background:**

Declining response proportions in surveys have been observed internationally. Improving response proportions is important for the generalizability of the outcome. The aim of this study was to examine the potential of animation videos to improve response proportions and sample composition in health surveys.

**Methods:**

A randomized trial was embedded in the Danish National Health Survey 2021 (n = 186,113) where the use of animation videos in the digital invitation letter was tested as a mean to increase response proportion. The effect of both demographic-targeted videos and a general video was tested. The sample was stratified into four subsamples; (1) individuals with non-western background and a non-Danish citizenship (n = 9,956), (2) men aged 16–24 years (n = 12,481), (3) women aged 75 years or older (n = 7,815) and (4) the remaining individuals (n = 155,861). The fourth subsample was randomized into two equal sized groups; a group receiving the general video and a control group receiving no video. Each of the first three subsamples was subsequently randomized into three subgroups with 25% receiving the target group video, 25% receiving the general video and 50% receiving no video. A total of four reminders (one digital and three postal) were sent to the eligible population.

**Results:**

The use of animation videos resulted in similar or slightly lower overall response proportion compared to the control group. The different animation videos were found to have heterogeneous effects on response proportions. A positive effect was found among men aged 16–24 years before the delivery of the postal reminder for the targeted animation video compared to no video (odds ratio: 1.13; 95% confidence interval: 1.02–1.26). Overall, the targeted animation videos tended to produce higher response proportions than the general animation video.

**Conclusions:**

The heterogeneous effects of the videos suggest that there is some potential for the use of animation videos to improve response proportions and sample composition. The content, target group and timing of evaluation seem to be important for the animation videos to be successful. This warrants further research to better identify in which contexts, in which subgroups and under which circumstances, animation videos are useful to increase response proportions.

**Trial registration:**

ClinicalTrials.gov ID: NCT05520242, registered 08/26/2022.

**Supplementary Information:**

The online version contains supplementary material available at 10.1186/s12874-023-02005-4.

## Background

Declining response proportions in health surveys have been observed internationally over the last decades [1]. The general decline in response proportions has raised concern among health survey researchers who attempt to obtain estimates from population-representative samples that are generalizable to the entire population. Consequently, survey design features affecting response proportions have been subject to extensive research [2–7]. Design features suggested to affect response proportions include e.g., incentives, use of prenotification, survey administration mode, the nature of the questionnaire (e.g., contents, length, design, layout and language), and use of reminders [3, 6]. Further, the literature shows that even subtle differences in design features can affect response proportions [2–5]. In general, response proportions can be stimulated by either a reduction in burden or an increase in motivation. Hence, an increased focus has been on targeted designs features as a mean to increased motivation for the individual sample member [2, 5].The invitation letter is the first information presented to the sample members upon invitation. Hence, modification in the invitation letter regarding motivational statements and level of complexity could be ways of improving response proportions.

The medium of video has many documented advantages for public health promotion compared to paper materials or spoken instructions. Video has been shown to be more effective in improving long-term knowledge retention [8], short-term recall [9] and message delivery [10] than paper materials or spoken instruction alone. Studies have found that animated videos specifically are an effective public health resource for knowledge transmission [10], improving health literacy [11] and brain health [12] and reducing anxiety [13]. These types of videos have been found to be relatable, entertaining and simple to understand, and previous video technology research has suggested the importance of enjoyment for supporting the acquisition of knowledge [14]. A previous study has examined the use of animation videos to recruit participants to a case-control study [15]. However, the use of animation videos as a mean to increase response proportions in large-scale health surveys have, to our knowledge, not previously been investigated.

The aim of this study was to examine the potential of animation videos to improve response proportions and sample composition in an embedded randomized controlled trial in the Danish National Health Survey 2021 (DNHS-2021). The overall research question was whether digital invitation letters including an animation video could produce higher response proportions compared to digital letters without an animation video. The proposition was that the inclusion of animation videos in the invitation letters should increase the willingness of sample members to participate by means of increased motivation and reduced complexity, and that this would be reflected in higher response proportions. In addition, the study also examines the effect of different versions of the animation videos (targeted animations videos and a general non-targeted animation video) on response proportions across sample subgroups.

## Methods

### Study population

The study was conducted as a randomized trial embedded in DNHS-2021, where the population was based on six mutually exclusive random subsamples among the adult (aged 16 years or older) Danish population: one in each of the five Danish administrative regions, and one national sample. The samples were drawn from the adult population in Denmark (including institutionalized people) using the Danish Civil Registration System which contains basic information on all people who are currently residing in Denmark [16]. No written or verbal consent is required according to Danish legislation. The randomized trial was conducted in the national sample (n = 25,000), the sample in the Capital Region (n = 102,500), the sample in Region Zealand (n = 34,000) and in the sample in the North Denmark Region (n = 39,700). The overall sample design and characteristics of the DNHS in the different years are described in detail elsewhere [17, 18].

### Data collection

The data collection was conducted from February 2021 to May 2021. All invitations were distributed in the first week of February. The majority (91.3%) of the DNHS sample was invited via a secure electronical mail service (Digital Post) to complete a web questionnaire. Individuals who were not registered to use Digital Post (8,7%) were sent an initial invitation letter via the regular postal service, inviting them to complete a web questionnaire or the enclosed paper questionnaire (mixed-mode contact). Thus, the DNHS sample in 2021 was comprised of two subsets based on the mode of initial contact: (a) individuals invited initially by Digital Post, and (b) individuals invited via the regular postal service only. The total study sample in the present study consisted of 201,200 individuals from original national sample, Capital Region, Region Zealand and North Denmark Region whereof 15,087 (7.5%) had unsubscribed digital post. Hence, the eligible study population receiving a digital invitation was 186,133 individuals. The proportion who was not registered to use Digital Post varied from 0.8% for the age group 16–24 years (men: 0.9%; women: 0.7%) to 35.8% for the age group 75 years or older (men: 27.9%; women: 41.7%).

**Fig. 1:**
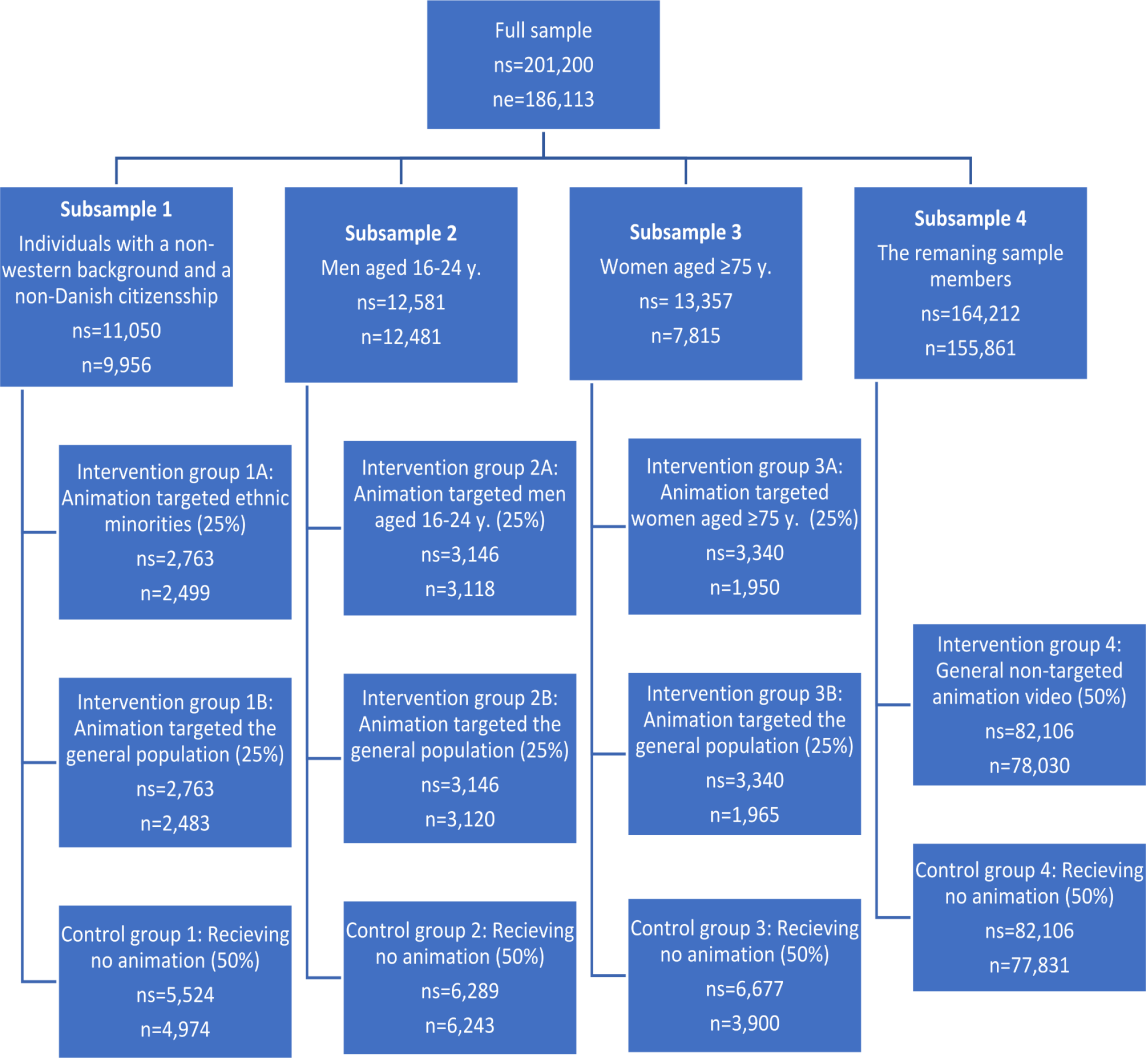
Illustration of the randomization into the control and intervention groups and sample size in the different groups (ns = number of individuals eligible for study; n = number of individuals in the different groups after exclusion of sample members who had unsubscribed digital post)

A total of four reminders, excluding the initial invitation letter, were sent to the eligible population. If the web questionnaire was not completed after one week, a digital reminder was sent to non-response individuals. After yet another two weeks of non-response, these individuals were approached by a reminder letter sent by regular postal service. Enclosed in this letter was a paper questionnaire with identical content to the web questionnaire and a pre-paid return envelope. The remaining two reminders were sent by regular postal service, the last one with an enclosed paper questionnaire and a pre-paid return envelope. An overview of the reminder procedure in the digital path is given in supplementary material. The animation videos were part of the first digital invitation letter as well as the digital reminder for those groups randomized to receive a video (see details about randomization below and Fig. 1).

### Development of the animation videos

**Fig. 2:**
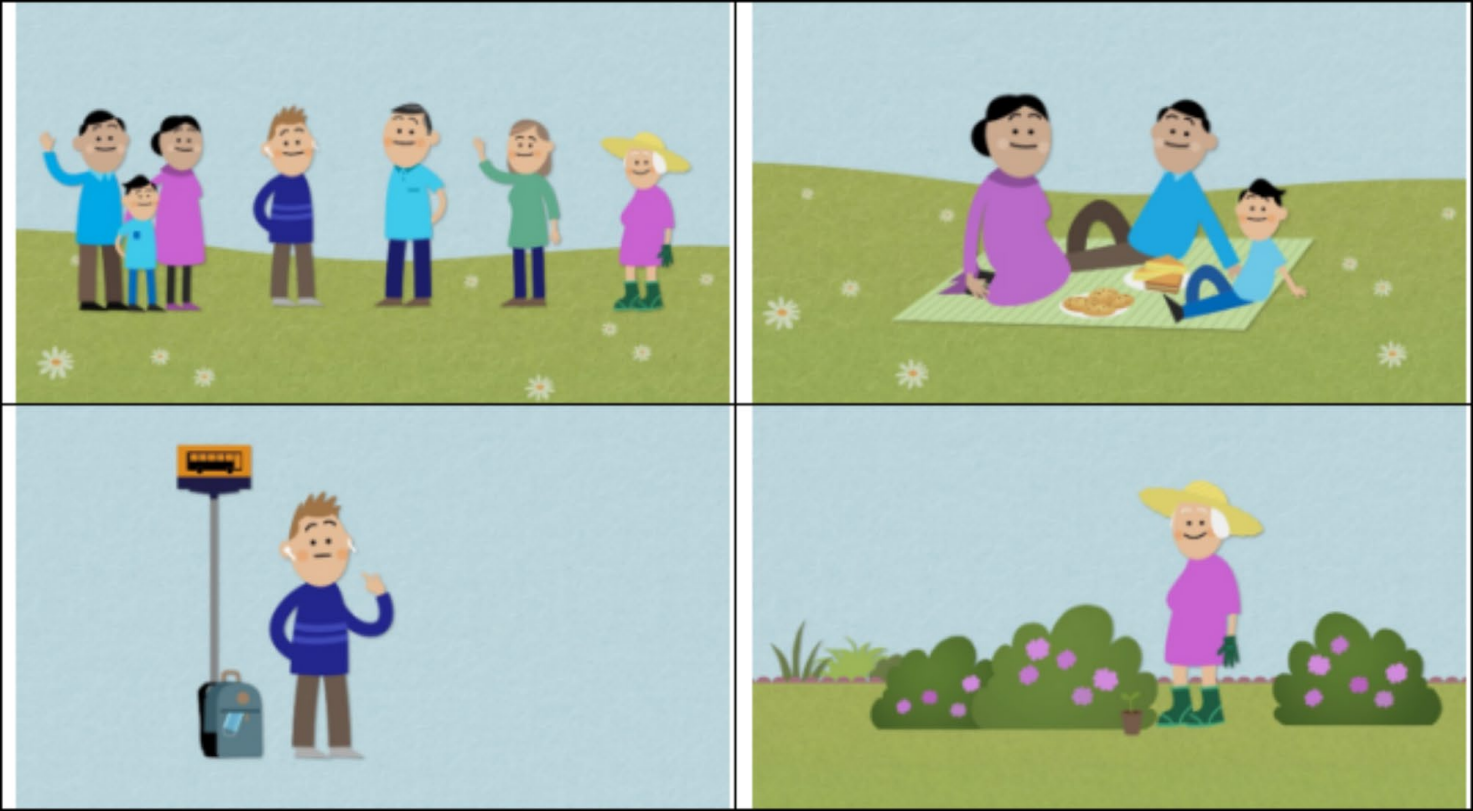
The still illustrations used in the digital invitation letter, i.e., general video (top left - the non-targeted video), ethnic minorities (top right – a targeted video), men aged 16–24 years (bottom left – a targeted video), and women aged 75 years or older (bottom right – a targeted video)

The animation videos were developed by the company *The Animation Workshop* (https://animationworkshop.via.dk/) in collaboration with the researchers conducting DNHS-2021, i.e., researchers from the National Institute of Public Health and the participating regions. First, a general non-targeted animation video was developed. The scientific base for the development was international literature describing design features known to affect participation – e.g., appeal to participate, use of incentives and perception of relevance [2, 5–7]. Further, existing knowledge in the research group gained from previous quantitative and qualitative work was included [19]. Hence, the videos included both an altruistic appeal mentioning how the results would be used by politicians in health care planning, and an egoistic appeal mentioning the possibility to win prizes upon participation and the importance of each respondent’s participation. To examine the effect of using targeted animation videos in different target groups, the general video was subsequently modified into three additional animation videos targeting young men, elderly women, and individuals with a non-western background and a non-Danish citizenship, respectively. Modification included changes in the animated persons in the videos, so they represented the target group, and slight changes in story line-wording, including mentioning that more answers were needed from their specific group (an English translation of the specific wording is given in supplementary material). Lastly, the Animation Workshop involved individuals from the different target groups in the further development and refinement of the videos. No additional pilot study was conducted. A version of the invitation letter with and without a still picture from one of the four animation videos was developed for digital distribution. The still picture included a play button and represented the first picture in the specific animation (Fig. 2). The animation video was presented in a new tap upon pressing the play button and with the following heading ‘Watch the movie about how we use your response’. The wording in the initial invitation letters including an animation video was identical to the generic invitation letter without an animation video. The placement of the animation video in the invitation letter is illustrated in supplementary material. Further, the animation videos are available as supplementary files.

### Categorization of target groups

The Danish Civil Registration System was used to retrieve information on sex, age, ethnic background, and marital status. This information was used to categorize sample members in four separate subsamples; (1) sample members with non-western background and a non-Danish citizenship, (2) men aged 16–24 years, (3) women aged 75 years or older and (4) the remaining sample members. The first 3 subsamples were known to have relatively low overall response proportions in previous waves of DNHS [17]. Ethnic background was defined using information on the respondents’ birthplace and citizenship and the parental birthplace. The following countries were categorized as western countries: The 27 European Union Member States, The United Kingdom, Andorra, Iceland, Liechtenstein, Norway, Monaco, San Marino, Switzerland, Vatican City, Australia, New Zealand, Canada, and the United States. All other countries were defined as non-Western countries. The following classification was applied: if both parents’ birthplaces were known, the country of origin was based on the birthplace of the mother, except if the father was born in Denmark, the mother was born abroad, and the respondent was a Danish citizen. If so, the country of origin was Denmark. If birthplace information was available for only one parent, that parent’s birthplace was used. When information for both parents was missing, the country of origin was defined from the respondent’s birthplace. If the respondent’s birthplace also was unknown, the country of origin was determined from the respondent’s citizenship.

### Randomization

Stratification into subsamples and their size as well as randomization of each subsample are illustrated in Fig. 1. Each of the first three subsamples (sample members with non-western background and a non-Danish citizenship, men aged 16–24 years and women aged 75 years or older, respectively) were subsequently randomized into three subgroups, with 25% receiving the target group video, 25% receiving the general non-targeted video and 50% receiving the generic letter with no video (i.e., a control group). For example, the subsample with non-western background and a non-Danish citizenship were divided into the following (a) a group receiving the video targeted individuals with non-western background and a non-Danish citizenship (25%) (b) a group receiving the general non-targeted video (25%) and (c) a control group receiving the generic letter with no video (50%). Subsample four (the remaining sample members) was randomized into two equal sized groups; (1) a group receiving the general non-targeted video and (2) a control group receiving the generic letter with no video.

The stratification and randomization were conducted by the National Institute of Public Health in collaboration with project members from the participating regions. Due to the timing of the different processes in conduction of the survey, the randomization was conducted before the initial split into (a) members registered to receive digital post and (b) members not registered to receive digital post. Hence, all eligible individuals were included in the randomization. As mentioned previously, it was only feasible to conduct the study among sample members who were registered to receive digital post. In the present study, 7.5% of the study sample were excluded as they were not registered to receive digital post. This exclusion is mainly an issue for subsample three (women aged 75 years or older), as the proportion who have unsubscribed digital post is high in this group (41.7%). The Fig. 1 shows both the number of eligible individuals in the different subsamples and groups and the final number of individuals after exclusion of individuals who have unsubscribed digital post. The randomization was conducted using the permuted blocks method.

### Statistics

By the end of data collection, data on both respondents and non-respondents, intervention and control groups etc. was stored inhouse and prepared for analysis. The response proportion was calculated as the number of respondents divided by the number of invited individuals before the delivery of the first postal reminder (day 22 from initial invitation) to prevent interference from postal respondents who have or may not have seen the digital invitation. Further, the final response proportion was calculated for all respondents by the end of the survey. Descriptive statistics and univariate logistic regression modelling of the 186,133 study participants were conducted to answer the research questions. In all analyses, the dependent variable indicates whether the sample member has (fully or partially) completed the questionnaire. In the first step, the independent variable was a dichotomous indicator of intervention (animation vs. no animation). This analysis will give an initial indication of whether, on average, the letters with animation videos have any overall effect on response propensity (Table 1). Second, we compared the effect of each targeted animation video vs. the general non-targeted video (Table 2). In the third step, we aimed to investigate whether any such overall effect of letters with animation videos is heterogenous across demographic subgroups and urbanization (Table 3). For the latter, Eurostat’s classification of urban and rural areas was used to group the 98 Danish municipalities into 3 types of areas: (a) cities, (b) towns and suburbs and (c) rural areas [20]. All analyses were carried out using SAS version 9.4.

## Results

On average, by day 22 from the initial invitation, the invitation letters including an animation video produced a slightly lower overall response proportion (37.7%) compared to the generic invitation letters without an animation video (38.5%) (OR: 0.97; 95% CI: 0.95–0.99) (Table 1). However, by the end of the survey, no significant difference was observed (OR: 0.99; 95% CI: 0.97–1.01). In general, a similar or slightly lower response proportion was observed in groups receiving an animation intervention (the different intervention groups) compared with the respective control groups (four control groups).However, in the intervention group (intervention group 2 A) consisting of men aged 16–24 years, the targeted animation video, produced a significantly higher response proportion (21.1%) by day 22 compared to the respective control group who got the generic invitation letter without a video (19.1%) (OR: 1.13; 95% CI: 1.02–1.26). By the end of the survey, no significant difference was observed (OR: 1.07; 95% CI: 0.98–1.17). In intervention group four (intervention group 4: the group receiving the non-targeted animation video to the general population), a negative effect on response proportion (39.9%), by day 22, was observed compared to the respective control group (40.6%) (OR: 0.97; 95% CI: 0.95–0.99). By the end of the survey, no significant difference was observed (OR: 0.99; 95% CI: 0.97–1.01). In the first intervention group (intervention group 1 A: Sample members with non-western background and a non-Danish citizenship) and the third intervention group (intervention group 3 A: Women aged 75 years or older), no significant differences were observed by day 22 (OR: 0.98; 95% CI: 0.86–1.11 and OR: 0.94; 95% CI: 0.84–1.05, respectively) or by the end of the survey (OR: 1.04; 95% CI: 0.94–1.16 and OR: 1.04; 95% CI: 0.91–1.18, respectively). compared to the respective control groups who got the generic invitation letter without a video.

**Table 1:** Response proportion (by day 22 and by the end of the survey, respectively) for intervention and control groups. Percent (descriptive) and OR (logistic regression)

Treatment group	Response proportion (%) – day 22	OR and 95% CI	Response proportion (%) – final	OR and 95% CI
**Overall**				
Animation (intervention)	37.7	0.97 (0.95–0.99)*	55.1	0.99 (0.97–1.01)
Generic letter / no animation (control)	38.5	Ref	55.4	Ref
**Subsample 1**				
Intervention group 1 A: Animation targeted ethnic minorities	17.0	0.98 (0.86–1.11)	30.9	1.04 (0.94–1.16)
Intervention group 1B: Animation targeted the general population (general video)	15.2	0.85 (0.75–0.97)*	29.0	0.96 (0.86–1.06)
Control group 1 (generic letter)	17.4	Ref	30.0	Ref
**Subsample 2**				
Intervention group 2 A: Animation targeted men aged 16–24 years	21.1	1.13 (1.02–1.26)*	36.0	1.07 (0.98–1.17)
Intervention group 2B: Animation targeted the general population (general video)	17.8	0.92 (0.82–1.03)	34.0	0.98 (0.90–1.07)
Control group 2 (generic letter)	19.1	Ref	34.5	Ref
**Subsample 3**				
Intervention group 3 A: Animation targeted women aged 75 years or older	52.4	0.94 (0.84–1.05)	76.2	1.04 (0.91–1.18)
Intervention group 3B: Animation targeted the general population (general video)	50.9	0.89 (0.80–0.99)*	73.8	0.92 (0.81–1.04)
Control group 3 (generic letter)	53.9	Ref	75.5	Ref
**Subsample4**				
Intervention group 4: Animation targeted the general population (general video)	39.9	0.97 (0.95–0.99)*	57.3	0.99 (0.97–1.01)
Control group 4 (generic letter)	40.6	Ref	57.7	Ref

The three targeted animation videos to specific subgroups tended to produce higher response proportions compared to the non-targeted animation video (Table 2). However, the difference was only statistically significant between the animation video targeted men aged 16–24 years and the general non-targeted animation video at day 22 (OR: 1.23; 95% CI: 1.09–1.40).

**Table 2:** Response proportion (by day 22 and by the end of the survey, respectively) and OR of response by animation video (targeted animation video vs. general animation video). Percent (descriptive) and OR (logistic regression)

Treatment group	Response proportion (%) – day 22	OR and 95% CI	Response proportion (%) – final	OR and 95% CI
**Subsample 1**				
Intervention group 1 A: Animation targeted ethnic minorities	17.0	1.15 (0.98–1.33)	30.9	1.09 (0.97–1.23)
Intervention group 1B: Animation targeted the general population (general video)	15.2	Ref	29.0	Ref
**Subsample 2**				
Intervention group 2 A: Animation targeted men aged 16–24 years	21.1	1.23 (1.09–1.40)*	36.0	1.09 (0.98–1.21)
Intervention group 2B: Animation targeted the general population (general video)	17.8	Ref	34.0	Ref
**Subsample 3**				
Intervention group 3 A: Animation targeted women aged 75 years or older	52.4	1.06 (0.94–1.20)	76.2	1.13 (0.98–1.31)
Intervention group 3B: Animation targeted the general population (general video)	50.9	Ref	73.8	Ref

Looking at the demographic variables and urbanization, generally no overall effect of the intervention with animation videos was seen on response proportion by the end of the survey in most subgroups (Table 3) The only exception was a slightly lower response proportion in the intervention group (sample members who received an animation video) among divorced (OR: 0.93; 95% CI: 0.88–0.98) and among individuals living in cities (OR: 0.97; 95% CI: 0.94–0.99) compared to the control group (sample members who didn’t receive an animation video).

**Table 3:** Overall Response proportion comparing intervention and control group by sociodemographic variables by the end of survey. Percent (descriptive) and OR (logistic regression)

		Animation Total population (N) and response proportion (%)	No animation Total population (N) and response proportion (%)	OR and 95%CI (animation vs. no animation)
Total		93,165 (55.1)	92,948 (55.4)	0.99 (0.97–1.01)
Sex	Men	46,189 (50.1)	(46,281) 50.3	0.99 (0.97–1.02)
	Women	46,976 (60.1)	46,667 (60.4)	0.99 (0.96–1.01)
Age	16–24 years	12,746 (40.7)	12,756 (40.9)	0.99 (0.94–1.04)
	25–44 years	29,125 (40.6)	29,056 (40.8)	0.99 (0.96–1.03)
	45–64 years	31,575 (60.5)	31,374 (60.9)	0.98 (0.95–1.01)
	≥ 65 years	19,719 (77.1)	19,762 (77.2)	1.00 (0.95–1.05)
Men	16–24 years	6,588 (34.2)	6,565 (33.8)	1.02 (0.95–1.09)
	25–44 years	14,513 (33.7)	14,637 (34.4)	0.97 (0.92–1.02)
	45–64 years	15,583 (55.6)	15,633 (55.9)	0.99 (0.94–1.03)
	≥ 65 years	9,505 (76.9)	9,446 (77.0)	1.00 (0.93–1.07)
Women	16–24 years	6,159 (47.7)	6,191 (48.5)	0.97 (0.90–1.04)
	25–44 years	14,612 (47.5)	14,419 (47.4)	1.01 (0.96–1.05)
	45–64 years	15,992 (65.3)	15.741 (65.9)	0.97 (0.93–1.02)
	≥ 65 years	10,214 (77.3)	10,316 (77.3)	1.00 (0.94–1.07)
Marital status	Married	43,347 (63.7)	43,484 (63.4)	1.01 (0.99–1.04)
	Divorced	10,570 (58.2)	10,432 (60.0)	0.93 (0.88–0.98)*
	Widowed	4,004 (71.2)	4,015 (70.9)	1.01 (0.92–1.12)
	Unmarried	35,244 (41.8)	35,017 (42.3)	0.98 (0.95–1.01)
Ethnic background	Danish	78,636 (58.7)	78,528 (58.9)	0.99 (0.97–1.01)
	Western	5,209 (40.5)	5,188 (40.8)	0.99 (0.91–1.07)
	Non-western	9.320 (33.3)	9,232 (33.4)	0.99 (0.94–1.06)
Urbanization	Cities	42,899 (52.7)	43,253 (53.5)	0.97 (0.94–0.99)*
	Towns and suburbs	14,973 (58.1)	14,668 (58.7)	0.97 (0.93–1.02)
	Rural areas	35,293 (56.8)	35,027 (56.3)	1.02 (0.99–1.05)

## Discussion

In this randomized trial, we examined whether the use of animation videos can increase participation in health surveys. At day 22 in the survey period, a slightly positive effect was observed for the animation video targeted men aged 16–24 years. When comparing the animation videos, the targeted animation videos tended to produce higher response proportions compared to the non-targeted animation video. However, only a significant difference was observed for men aged 16–24 at day 22. However, in general, a similar or slightly lower response proportion was observed in the intervention group who received an invitation letter including an animation video compared to the control group who received the generic invitation letter without an animation video. This was seen both at day 22 and by the end of the survey. When looking at the effect of the animation videos in different demographic subgroups, a slightly lower response proportion was observed in the intervention group among divorced and among sample members living in cities compared to the control group. Overall, both negative and positive effects of targeted animation videos was observed, and future surveys researcher should carefully select the target group and content in the video to gain a positive effect on response proportion and sample composition.

Previous studies have documented that the use of videos has advantages for public health promotion compared to paper materials or spoken instructions [8–10]. Further, studies have found that animated videos are an effective public health resource for e.g. knowledge transmission [10], and animated videos have been found to be relatable, entertaining and simple to understand [14]. A previous study found that an animated decision aid did not lead to greater intention to take part in a web-based case-control study [15]. This is in line with the findings in the present study. The lack of significant effect on response proportion in our study suggests that the content of the animation videos either did not increase motivation sufficiently for them to respond, that the animation videos were not actually watched by the receivers, or that the animation videos unintentionally distracted the sample members from the main task given in the invitation letter, i.e., completing the questionnaire, by asking them to watch the video and afterwards navigate back to the letter to access the survey. However, the heterogeneous effects of the findings suggest that there is potential for targeted animation videos to improve response proportions and sample composition for some groups. E.g., in the present study, targeted animation had a positive effect on the response proportion among men aged 16–24 years, which is the group with the lowest response proportion in earlier rounds. It is worth considering why the video had a positive effect in this group and not in the other subgroups. A possibly explanation could be that this group is better at navigating digitally; and thus, to a higher degree managed to both watch the video and navigate back to the letter to access the survey. Another possibility is that the content of the specific video was more adapt and motivating to this group than to the other groups. Hence, the use of targeted animation in this group has the potential to improve sample composition.

The major strength of the present study was the incorporation of a randomized trial in a large nationally population sample. This made is it possible to study the effect of animation videos in a real-life setting based on a highly scientific method. The study thereby adds to the scientific knowledge about the use of animation videos as a mean to reduce nonresponse in health surveys. Further, the large sample size made it possible to study the effect of both targeted and non-targeted animation videos. Yet, in cannot be rejected that lack of statistical power is the reason for the non-significant findings in subgroups. The study also had some methodological limitations. First, 7.5% of the study sample were excluded as they were not registered to receive digital post, with the majority being in the age group 75 years or older. The criteria for unsubscribing Digital Post include low technical skills and mental or physical disability [21]. Hence, this might obscure the effect estimates. In addition, it is unknown whether individuals randomized to receive the animation video actually watched the video. Maybe they intentionally or unintentionally missed the link in the invitation letter, or the animation video had the unintentional effect, that the sample members got distracted from the main task, i.e., completing the questionnaire. Either way, this might have obscured a potential effect. Further, multiple comparison was conducted in the study and some significant associations may have occurred even if there was no real difference, e.g., the significantly lower response proportion among divorced might be an example of this. Further, even though a lot of work and effort was put into the development of the animation videos, the content and style might not be the most suitable and effective. It is possible that the animation videos need to be even more targeted and refined in their content to the specific groups to improve response proportion. E.g., the study group including individuals with a non-western background and a non-Danish citizenship is a very diverse end heterogenic group. Hence, diverse effects in different cultural groups might block out an overall positive effect of the animation videos. However, this study provides a solid knowledge base which can be used as an important stepping-stone for future studies. Overall, the specific targeted designs that are suggested by the findings of this exploratory study should be tested in future confirmatory studies. Finally, there may be a potential economic gain in large scale studies produced by the higher response proportion among young men, which results in fewer postal reminder letters, compared to the cost for development and distribution of animation videos.

## Conclusion

In general, invitation letters with animation videos intended to increase response-motivation resulted in a similar or lower response proportion overall compared to a generic invitation letter without a video, hence no overall improvement in sample composition was reached. However, a positive effect on the response proportion was seen for the animation video targeted men aged 16–24 years compared to the generic invitation letter. Further, the three targeted animation videos tended to produce higher response proportions compared to the general non-targeted animation video. The heterogeneous findings suggest that there is potential for targeted animation videos to improve both response proportions and sample composition. The content of the video, the target group and timing of evaluation seem to be important for the use of targeted animation videos to be successful. This warrants further research to better identify in which contexts, in which subgroups and under which circumstances, animation videos are useful to increase response proportions.

### Electronic supplementary material

Below is the link to the electronic supplementary material.

Supplementary Material 1

Supplementary Material 2

Supplementary Material 3

Supplementary Material 4

Supplementary Material 5

Supplementary Material 6

Supplementary Material 7

## Data Availability

The datasets used and/or analysed during the current study are available from the corresponding author on reasonable request.
