# Supplementary material for: Do animation videos increase participation in national health surveys? A randomised controlled trial
Source: BMC Med Res Methodol. 2023 Aug 14;23:184. doi: 10.1186/s12874-023-02005-4 (PMC10424421; doi:10.1186/s12874-023-02005-4)
Supplement: Supplementary file 1 — Supplementary Material 1 [file 12874_2023_2005_MOESM1_ESM.pdf]

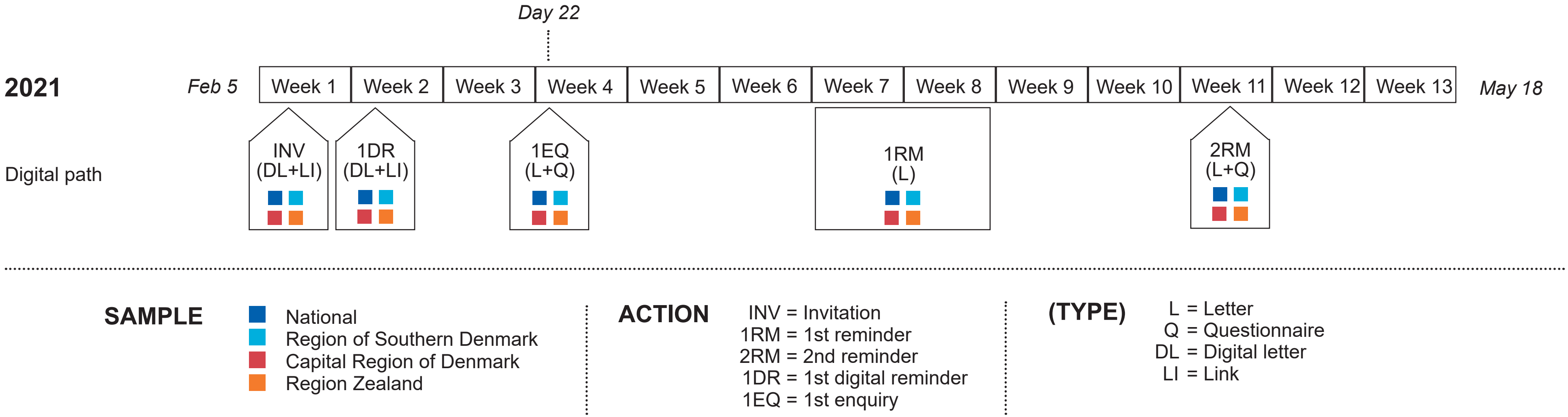

Day 22
2021
Feb 5
Week 1
Week 2
Week 3
Week 4
Week 5
Week 6
Week 7
Week 8
Week 9
Week 10
Week 11
Week 12
Week 13
May 18
Digital path
INV (DL+LI)
1DR (DL+LI)
1EQ (L+Q)
1RM (L)
2RM (L+Q)
SAMPLE
National
Region of Southern Denmark
Capital Region of Denmark
Region Zealand
ACTION
INV = Invitation
1RM = 1st reminder
2RM = 2nd reminder
1DR = 1st digital reminder
1EQ = 1st enquiry
(TYPE)
L = Letter
Q = Questionnaire
DL = Digital letter
LI = Link
