# Supplementary material for: Do animation videos increase participation in national health surveys? A randomised controlled trial
Source: BMC Med Res Methodol. 2023 Aug 14;23:184. doi: 10.1186/s12874-023-02005-4 (PMC10424421; doi:10.1186/s12874-023-02005-4)
Supplement: Supplementary file 2 — Supplementary Material 2 [file 12874_2023_2005_MOESM2_ESM.docx]

**General animation video**

Hi,

You are invited to participate in the large survey on the health of Danes. , and it is actually quite easy.

Just click on the link in the letter you received in your digital mailbox and you're good to go.

The survey is nationwide, and we conduct it every four years.

The results we collect are used by politicians in the formulation of health legislation. For the benefit of the entire population. And that's why your answer is important to us.

It takes about 45 minutes to complete the questionnaire. We also draw lots for great prizes among those who respond. Thank you for helping us helping Denmark.

**Animation video targeted ethnic minorities.**

Hi,

You are invited to participate in the large survey on the health of Danes. We can see from previous studies that we are missing answers from Danes of non-Danish ethnic origin.

It is actually quite easy. Just click on the link in the letter you received in your digital mailbox and you're good to go.

The survey is nationwide, and we conduct it every four years.

The results we collect are used by politicians in the formulation of health legislation. For the benefit of the entire population. And that's why your answer is important to us.

It takes about 45 minutes to complete the questionnaire. We also draw lots for great prizes among those who respond. Thank you for helping us helping Denmark.

**Animation video targeted women aged 75 years or older.**

Hi,

You are invited to participate in the large survey on the health of Danes. We can see from previous studies that we are missing answers from especially women aged 75 or older.

It is actually quite easy. Just click on the link in the letter you received in your digital mailbox and you're good to go.

The survey is nationwide, and we conduct it every four years.

The results we collect are used by politicians in the formulation of health legislation. For the benefit of the entire population. And that's why your answer is important to us.

It takes about 45 minutes to complete the questionnaire. We also draw lots for great prizes among those who respond. Thank you for helping us helping Denmark.

**Animation targeted men aged 16-24 years.**

Hi, we need you. Yes, you.

We're doing a large survey of how you're doing out there.

And we need answers from young men between 16-24, just like you. It is actually quite easy. Just click on the link in the letter you received in your digital mailbox and you're good to go.

The survey is nationwide, and we conduct it every four years.

The results we collect are used by politicians in the formulation of health legislation. For the benefit of the entire population. And that's why your answer is important to us.

It takes about 45 minutes to complete the questionnaire. We also draw lots for great prizes among those who respond. Thank you for helping us helping Denmark.
