# Supplementary material for: Do animation videos increase participation in national health surveys? A randomised controlled trial
Source: BMC Med Res Methodol. 2023 Aug 14;23:184. doi: 10.1186/s12874-023-02005-4 (PMC10424421; doi:10.1186/s12874-023-02005-4)
Supplement: Supplementary file 7 — Supplementary Material 7 [file 12874_2023_2005_MOESM7_ESM.pdf]

SDU
STATENS INSTITUT FOR FOLKESUNDHED

Complete the questionnaire
Win great prizes

Name / Adress

HOW ARE YOU DOING?

Help us learn

Copenhagen 5/2-2021

Dear XXXX

You are randomly selected to participate in a questionnaire survey on well-being, health and illness in Denmark. The study is part of a nationwide survey to be used to gain knowledge about the state of health in your municipality, in the region and throughout Denmark.

We need your answer

Your answer is important – whether you are healthy, less well or ill. The survey is used in the planning of actions that can improve public health.

We therefore hope that you will take the time to complete the questionnaire, but participation is of course voluntary. All responses are treated confidentially by the people working with the information, and the results are published in such a way that you cannot be recognized.

It is of great importance for the quality of the survey that as many people as possible choose to participate. That's why we send reminders to anyone from whom we haven't received a response.

Further information on the study can be found in the attached appendix or obtained from 6550 7717 (Monday-Wednesday and Friday 9am-12pm and Thursday 1pm-4pm) or by email: svar2021@si-folkesundhed.dk. You can also learn more about the study at www.sdu.dk/sif.

Your response is important to us and we appreciate the time you spend answering the questionnaire.

Thank you very much in advance for your participation.

Sincerely,

Morten Grønbæk
Director

Anne Illemann Christensen
Projektleader

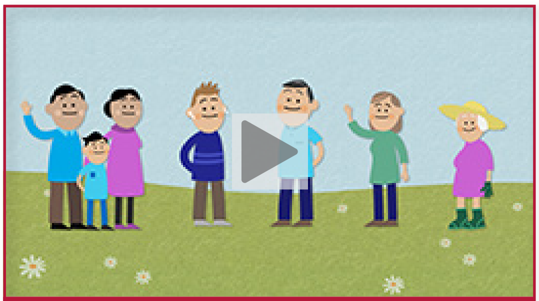

Watch the video on how we use your answer

Find the questionnaire by clicking here

If the questionnaire does not open automatically, you can fill it out at www.svar2021.dk, where you must use the following personal user name and password:
Username: XXXXX
Password: XXXXX
Your answer is important, it takes about 45 minutes to answer.

We draw lots for prizes from the forms received as a thank you for participating. Among all the entries, lots will be drawn for three cash prizes of DKK 5,000, DKK 3,000 and DKK 1,000, respectively.
Do you answer the questionnaire before On 15/2-2021, you are also in the draw for DKK 2,000. Winners will be notified directly by post.
